# Therapeutic Effects of a Traditional Chinese Medicine Formula Plus Tamoxifen vs. Tamoxifen for the Treatment of Mammary Gland Hyperplasia: A Meta-Analysis of Randomized Trials

**DOI:** 10.3389/fphar.2018.00045

**Published:** 2018-02-02

**Authors:** Hao-Tian Li, Hong-Hong Liu, Yu-Xue Yang, Tao Wang, Xue-Lin Zhou, Yang Yu, Su-Na Li, Yi Zheng, Ping Zhang, Rui-Lin Wang, Jian-Yu Li, Shi-Zhang Wei, Kun Li, Peng-Yan Li, Li-Qi Qian

**Affiliations:** ^1^Department of Pharmacy, 302 Military Hospital of China, Beijing, China; ^2^International Center for Liver Disease Treatment, 302 Military Hospital of China, Beijing, China; ^3^College of Pharmacy, Chengdu University of Traditional Chinese Medicine, Chengdu, China; ^4^Department of Traditional Chinese Medicine, First Affiliated Hospital of Chinese PLA General Hospital, Beijing, China; ^5^Integrative Medical Center, 302 Military Hospital of China, Beijing, China; ^6^China Military Institute of Chinese Medicine, 302 Military Hospital of China, Beijing, China

**Keywords:** mammary gland hyperplasia, Ru-Pi-Xiao, tamoxifen, efficacy and safety, hormonal parameters, breast lump, meta-analysis

## Abstract

As a common disorder that accounts for over 70% of all breast disease cases, mammary gland hyperplasia (MGH) causes a severe problem for the quality of patients' life, and confers an increased risk of breast carcinoma. However, the etiology and pathogenesis of MGH remain unclear, and the safety and efficacy of current western drug therapy for MGH still need to be improved. Therefore, a meta-analysis was conducted by our team to determine whether a TCM formula named Ru-Pi-Xiao in combination with tamoxifen or Ru-Pi-Xiao treated alone can show more prominent therapeutic effects against MGH with fewer adverse reactions than that of tamoxifen. Studies published before June 2017 were searched based on standardized searching rules in several mainstream medical databases. A total of 27 articles with 4,368 patients were enrolled in this meta-analysis. The results showed that the combination of Ru-Pi-Xiao and tamoxifen could exhibit better therapeutic effects against MGH than that of tamoxifen (OR: 3.79; 95% CI: 3.09–4.65; *P* < 0.00001) with a lower incidence of adverse reactions (OR: 0.35; 95% CI: 0.28–0.43; *P* < 0.00001). The results also suggested that this combination could improve the level of progesterone (MD: 2.22; 95% CI: 1.72–2.71; *P* < 0.00001) and decrease the size of breast lump (MD: −0.67; 95% CI: −0.86 to −0.49; *P* < 0.00001) to a greater extent, which might provide a possible explanation for the pharmacodynamic mechanism of Ru-Pi-Xiao plus tamoxifen. In conclusion, Ru-Pi-Xiao and related preparations could be recommended as auxiliary therapy combined tamoxifen for the treatment of MGH.

## Introduction

Mammary gland hyperplasia (MGH), also known as hyperplastic disease in breast, refers to the lesions of mammary ducts and lobules. Patients with MGH can feel pain in their breasts, accompanied by the formation of breast lumps. As a common disorder occurring more frequently in young and middle-aged females, MGH accounts for over 70% of all breast disease cases, seriously affecting normal lives of these patients (Cowin and Wysolmerski, 2010; Su, 2012).

Nowadays, the etiology and pathogenesis of MGH have not yet been fully understood. However, it has become a generally accepted view that the increased level of estrogen secretion could induce the up-regulation of plasma estradiol concentration and inadequate production of progesterone, leading to the duct ectasia of breast and cyst formation, and then cause proliferation in breast tissue (Coussens and Pollard, 2011; Arendt and Kuperwasser, 2015).

The pathogenesis of MGH is similar to that of breast cancer. In recent years, more and more attention has been paid to the carcinogenesis tendency of MGH. It has been reported by the Union for International Cancer Control (UICC) that MGH could increase the risk of breast cancer. In the United States, the incidence of breast carcinogenesis in women suffering from MGH is nearly twice that of the ordinary female population (Jin, 2002). Thus, the treatment of MGH is believed to be an effective approach for breast cancer prevention (Ercan et al., 2011; Visscher et al., 2017).

In the treatment of MGH with western medicine, pharmacists often use hormone preparations (e.g., androgen and progesterone), hormone receptor inhibitors (like tamoxifen) and prolactin inhibitors (e.g., bromocriptine, danazol, and iodine preparations) as therapeutic drugs (Huo and Liu, 2015). Among them, tamoxifen is the most commonly used drug. As an estrogen antagonist that competes with estrogen to bind to the estrogen receptors of breast cells, tamoxifen directly blocks estrogen-mediated effects, and thereby improves the clinical symptoms of MGH. Although, tamoxifen is widely prescribed to treat MGH, the safety and efficacy of tamoxifen for MGH are still not good enough.

In China, TCM compounds have been extensively accepted and utilized in the treatment of MGH (Li et al., 2017). For instance, Ru-Pi-Xiao is a kind of TCM formula most widely used for treating MGH, which is composed of antlers, dandelion, kelp, radix paeoniae rubra, carthamus tinctorius, etc. Extracts from antlers show anti-platelet aggregation effects on MGH. Dandelion-derived Compounds like taraxacin are proved to have immunomodulatory activity. Kelp, rich in iodine element, can improve luteal function, increase the absorption of pathological products and inflammatory exudates in breast tissue, and then exhibit therapeutic efficacy on MGH. The combination of radix paeoniae rubra and carthamus tinctorius can also exert anti-platelet aggregation activities, and promote fibrinolysis in the treatment of MGH. Studies have indicated that the combined use of Ru-Pi-Xiao and tamoxifen may show prominent therapeutic effects against MGH with a low incidence of adverse reactions. Nevertheless, the pharmacodynamic mechanism of Ru-Pi-Xiao remains to be further revealed.

Therefore, the objective of this systematic review was to assess the evidences regarding the effectiveness and safety of Ru-Pi-Xiao in the treatment of MGH, and clarify whether Ru-Pi-Xiao in combination with tamoxifen or Ru-Pi-Xiao treated alone could relieve MGH symptoms and improve hormonal parameters (e.g., plasma estradiol, progesterone, and lutrophin).

## Methods

This meta-analysis was performed and reported in accordance with the Preferred Reporting Items for Systematic Reviews and Meta Analyses (PRISMA) guidelines.

### Database and search strategies

We searched for relevant reports published from the electronic data sources as follows: PubMed (1961–2017), EMBASE (1990–2017), Wiley Online Library (1999–2017), Springer link (1978–2017), China Knowledge Resource Integrated database (1915–2017), SinoMed database (1978–2017), Wanfang database (1998–2017), and VIP database for Chinese Technical Periodicals (1989–2017). The journal languages were restricted to Chinese and English, and the literature search was constructed around terms for Mammary Gland Hyperplasia, Ru-Pi-Xiao (or Rupixiao), and tamoxifen.

### Inclusion criteria

#### Types of studies

All reports included in this article were for clinical randomized controlled trials (RCTs).

#### Types of participants

According to the “Criteria of Diagnosis and Therapeutic Effect of Diseases and Syndromes in Traditional Chinese Medicine (1994)” created by State Administration of Traditional Chinese Medicine, all subjects included in this review had been diagnosed with MGH (“Ru Xian Zeng Sheng” in Chinese) by means of color Doppler sonography, mammography and physical examination. Patients with breast cancer, breast fibroma, serious organ dysfunction, or immune system diseases were excluded, as well as patients in pregnancy and lactation. Subject sex was restricted to female. Ages and disease duration of subjects were unrestricted.

#### Types of interventions

Trials were divided into treatment group and control group based on the intervention methods, with the experimental group receiving the combination of Ru-Pi-Xiao and tamoxifen, and the control group receiving tamoxifen alone. The dosages of tamoxifen in the experimental group were the same as that in the control group.

#### Types of outcome measures

Researches were eligible if they assessed at least one of the following outcome measures: overall response rate of MGH, level of plasma estradiol, level of progesterone, level of lutrophin, and diameter of breast lumps. If available, safety was defined as the number of adverse events that occurred during the studies.

### Data extraction and management

Three authors independently used the same selection criteria to screen titles, abstracts, and contexts of the relevant studies. The reports that failed to meet the inclusion criteria were excluded. Any disagreement was resolved by discussion, and a third author would be consulted in the case of persisted disagreement. Then, data were extracted from the selected articles, which included study characteristics (e.g., author and year), participant characteristics (e.g., age, sample size), disease course, intervention and dosage, duration of treatment, and outcome measures.

### Quality assessment

Three reviewers independently assessed the quality of included studies using Cochrane Collaboration's tool to analyze the risk of bias. The following information was evaluated: random allocation, concealed allocation, blind fashion, and reporting biases. Disagreements between reviewers were resolved through discussion.

### Statistical analysis

Meta-analysis was conducted using Review Manager software (version 5.3). Odds ratio (OR) with 95% confidence intervals (CI) was reported for the dichotomous data, and mean differences (MD) with 95% CI for the continuous data. Statistical heterogeneity between studies was tested by calculating Higgins *I*^2^ values or using the Chi-square test. *I*^2^ > 25 %, *I*^2^ > 50%, and *I*^2^ > 75% were, respectively defined to indicate moderate, substantial, and considerable heterogeneity. When the *P*-value of this test was <0.1, an *I*^2^ test was carried out. If the *I*^2^ test showed a value >50%, a random effects model was carried out. Otherwise, a fixed effects model was carried out. A *P-*value lower than 0.05 was considered to be statistically significant.

## Results

### Study selection

To evaluate the therapeutic effects of Ru-Pi-Xiao and tamoxifen for MGH, a total of 768 records were identified from eight Chinese and English databases. After the duplicates were removed, 429 potentially relevant abstracts were initially screened, and 385 were excluded by analyzing the abstract. Seventeen were excluded after assessing the full text, due to the lack of “Ru-Pi-Xiao Plus Tamoxifen” treated group (Fan, 2005; Lv et al., 2006; Li and Li, 2008; Liu et al., 2008; Bai, 2010; Li and Zhang, 2010; Liu, 2010; Xing et al., 2011; Tang, 2012; Wang et al., 2012; Yuan M., 2013; Yuan X. Y., 2013; Dao, 2015; Ouyang, 2015; Pu, 2015; Gao and Li, 2016; Xiao and Yang, 2016).

**Figure 1:**
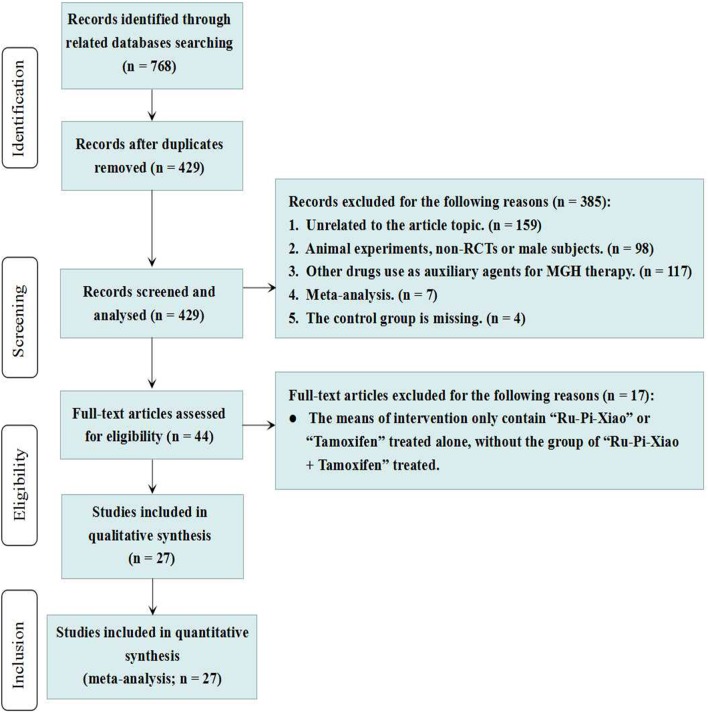
Flowchart of the results of the literature search. MGH, mammary gland hyperplasia.

Finally, 27 full-text articles met our inclusion criteria (Xia and Deng, 2001; Wang et al., 2004, 2014, 2015; Yang, 2006; Ma and Xu, 2007; Liu et al., 2008, 2013; Li, 2009, 2014; Zheng, 2011; Zhang et al., 2012, 2013; Huang and Yi, 2014; Wang, 2014, 2015; Xiao et al., 2014; Liu, 2015; Yin et al., 2015; Yue, 2015; Zhao, 2015; Cao, 2016; Huang, 2016; Kong and Huang, 2016; Ren, 2016; Xia, 2016; Zhu, 2016). All RCTs included in this meta-analysis were conducted in China and published in Chinese (the flowchart of 27 RCTs included is indicated in Figure 1).

### Characteristics of included studies

A total of 4,368 female participants (18–62 years old) were included in eligible RCTs, which were conducted between 2001 and 2016. The characteristics of the included studies, including author and year, sample size, age, disease course, intervention and dosage, duration of treatment, and outcome measures, are presented in Table 1. In 20 of the included reports, participants were divided into two groups, which were “experimental group” (abbreviated to “E” in Table 1; these subjects were treated with Ru-Pi-Xiao plus tamoxifen) and “control group” (abbreviated to “C” in Table 1; these subjects were treated with tamoxifen alone), while patients were classified into three groups in seven other included papers, including “experimental group 1” (abbreviated to “E1” in Table 1; these subjects were treated with Ru-Pi-Xiao plus tamoxifen), “experimental group 2” (abbreviated to “E2” in Table 1; these subjects were treated with Ru-Pi-Xiao alone), and “control group” (abbreviated to “C” in Table 1; these subjects were treated with tamoxifen alone).

**Table 1:** Characteristics of the included studies.

**Author, year**	**Sample size**	**Age(year)**	**Disease course**	**Intervention, dosage**	**Duration**	**Outcome measures**
Zhang et al., 2013	E1: 81 E2: 81 C: 81	E1: 40.3 ± 7.1 years E2: 41.2 ± 6.8 years C: 39.6 ± 6.5 years	E1: 35.1 ± 10.3 months E2: 35.3 ± 9.8 months C: 34.8 ± 9.6 months	E1: Ru-Pi-Xiao(3.84 g/d) + Tamoxifen(20 mg/d) E2: Ru-Pi-Xiao(3.84 g/d) C: Tamoxifen(20 mg/d)	3 months	(a) The total effective rates (b) The level of plasma estradiol (c) The level of progesterone (d) The level of lutrophin (e) Side effects after treatment
Wang, 2015	E:50 C:50	32.4 ± 6.8 years	–	E: Ru-Pi-Xiao(6 g/d) + Tamoxifen(20 or 40 mg/d) C: Tamoxifen(20 or 40 mg/d)	–	(a) The total effective rates (e) Side effects after treatment
Cao, 2016	E:48 C:48	E: 41.58 ± 2.21 years C: 41.45 ± 2.14 years	E: 3.67 ± 0.35 years C: 3.58 ± 0.36 years	E: Ru-Pi-Xiao(9 tablets) + Tamoxifen(20 mg/d) C: Tamoxifen(20 mg/d)	3 months	(a) The total effective rates (b) The level of plasma estradiol (c) The level of progesterone (d) The level of lutrophin (f) Diameter of breast lumps
Wang et al., 2014	E:40 C:40	E: 36.3 ± 2.8 years C: 35.6 ± 2.1 years	E: 4.1 ± 1.5 years C: 3.2 ± 1.1 years	E: Ru-Pi-Xiao(12 tablets/d) + Tamoxifen(20 mg/d) C: Tamoxifen(20 mg/d)	3 months	(a) The total effective rates (b) The level of plasma estradiol (c) The level of progesterone (d) The level of lutrophin (e) Side effects after treatment
Ma and Xu, 2007	E1: 54 E2: 52 C: 52	E1: 18–55 years E2: 19–50 years C: 20–50 years	E1: 3 months-10 years E2: 2 months-9 years C: 2 months-10 years	E1: Ru-Pi-Xiao(15 tablets/d) + Tamoxifen(10 mg/d) E2: Ru-Pi-Xiao(15 tablets/d) C: Tamoxifen(10 mg/d)	3 months	(a) The total effective rates (e) Side effects after treatment
Li, 2014	E:60 C:60	19–54 years	1 months-4 years	E: Ru-Pi-Xiao(4.8 g/d) + Tamoxifen(20 mg/d) C: Tamoxifen(20 mg/d)	3 months	(a) The total effective rates
Liu, 2015	E:26 C:28	E: 36.6 ± 4.8 years C: 35.9 ± 3.7 years	–	E: Ru-Pi-Xiao(15 tablets/d) + Tamoxifen(20 mg/d) C: Tamoxifen(20 mg/d)	3 months	(a) The total effective rates (e) Side effects after treatment (f) Diameter of breast lumps
Huang and Yi, 2014	E:30 C:30	E: 36.5 ± 2.5 years C: 38.5 ± 2.5 years	E: 1 months-7 years C: 2 months-6 years	E: Ru-Pi-Xiao(5.1 g/d) + Tamoxifen(20 mg/d) C: Tamoxifen(20 mg/d)	3 months	(a) The total effective rates (b) The level of plasma estradiol (c) The level of progesterone (d) The level of lutrophin (e) Side effects after treatment (f) Diameter of breast lumps
Ren, 2016	E:51 C:51	E: 39.6 ± 2.9 years C: 39.2 ± 2.7 years	–	E: Ru-Pi-Xiao(15 tablets/d) + Tamoxifen(20 mg/d) C: Tamoxifen(20 mg/d)	—	(a) The total effective rates
Liu et al., 2013	E:128 C:128	E: 36.45 ± 8.42 years C: 36.79 ± 7.64 years	E: 16.14 ± 7.23 months C: 15.27 ± 8.39 months	E: Ru-Pi-Xiao(6 g/d) + Tamoxifen(20 mg/d) C: Tamoxifen(20 mg/d)	3 months	(a) The total effective rates
Huang, 2016	E:57 C:57	36.5 ± 5.2 years	25 ± 11 months	E: Ru-Pi-Xiao(3 bags/d) + Tamoxifen(10 mg/d) C: Tamoxifen(10 mg/d)	1 months	(a) The total effective rates
Zhu, 2016	E:43 C:43	E: 42.0 ± 1.1 years C: 42.0 ± 1.2 years	–	E: Ru-Pi-Xiao(12 tablets/d) + Tamoxifen(20 mg/d) C: Tamoxifen(20 mg/d)	3 menstrual cycle	(a) The total effective rates (e) Side effects after treatment
Xiao et al., 2014	E1: 54 E2: 50 C: 52	18–54 years, Average Age: 30.5 years	2 months-10 years, Average course: 2.5 years	E1: Ru-Pi-Xiao(1.28 g/d) + Tamoxifen(20 mg/d) E2: Ru-Pi-Xiao(1.28 g/d) C: Tamoxifen(20 mg/d)	3 menstrual cycle	(a) The total effective rates (e) Side effects after treatment
Wang, 2014	E:60 C:60	E: 38.1 ± 1.2 years C: 38.0 ± 1.1 years	–	E: Ru-Pi-Xiao(12 tablets/d) + Tamoxifen(20 mg/d) C: Tamoxifen(20 mg/d)	3 months	(a) The total effective rates (e) Side effects after treatment
Zhang et al., 2012	E:63 C:63	38.2 ± 5.3 years	32 ± 9 months	E: Ru-Pi-Xiao(12 tablets/d) + Tamoxifen(20 mg/d) C: Tamoxifen(20 mg/d)	3 menstrual cycle	(a) The total effective rates (b) The level of plasma estradiol (c) The level of progesterone (d) The level of lutrophin (e) Side effects after treatment
Wang et al., 2015	E:50 C:50	25–55 years	4 months-7 years	E: Ru-Pi-Xiao(12 tablets/d) + Tamoxifen(20 mg/d) C: Tamoxifen(20 mg/d)	3 months	(a) The total effective rates (b) The level of plasma estradiol (c) The level of progesterone (d) The level of lutrophin
Yue, 2015	E:56 C:56	38.1 ± 3.6 years	32.4 ± 8 months	E: Ru-Pi-Xiao(12 tablets/d) + Tamoxifen(20 mg/d) C: Tamoxifen(20 mg/d)	3 months	(a) The total effective rates (b) The level of plasma estradiol (c) The level of progesterone (d) The level of lutrophin (e) Side effects after treatment
Xia and Deng, 2001	E1: 58 E2: 58 C: 52	30.5 ± 10.45 years	1 months-12 years, Average course: 2 years and 1 month	E1: Ru-Pi-Xiao(12 pills/d) + Tamoxifen(20 mg/d) E2: Ru-Pi-Xiao(12 pills/d) C: Tamoxifen(20 mg/d)	2 or 4 menstrual cycle	(a) The total effective rates (e) Side effects after treatment
Kong and Huang, 2016	E:93 C:93	E: 35.5 ± 3.9 years C: 36.5 ± 4.7 years	E: 15.18 ± 7.32 months C: 16.25 ± 7.31 months	E:Ru-Pi-Xiao(9 tablets/d)+Tamoxifen(20–40 mg/d) C:Tamoxifen(20–40 mg/d)	–	(a) The total effective rates (e) Side effects after treatment
Liu et al., 2008	E:181 C:165	Average Age: 35.2 years	7 days-12 years	E: Ru-Pi-Xiao(12 pills/d) + Tamoxifen(First month: 20 mg/d; Second month: 10 mg/d) C: Tamoxifenn(First month: 20 mg/d; Second month: 10 mg/d)	2–3 months (Tamoxifen); 45 d (Ru-Pi-Xiao)	(a) The total effective rates (e) Side effects after treatment
Yin et al., 2015	E:75 C:75	E: 20–65 years C: 22–60 years	E: 2 months-8 years C: 3 months-7 years	E: Ru-Pi-Xiao(6 g/d) + Tamoxifen(20 mg/d) C: Tamoxifen(20 mg/d)	3 months	(a) The total effective rates (e) Side effects after treatment
Zhao, 2015	E:72 C:72	23–62 years	3–8 months	E: Ru-Pi-Xiao(6 g/d) + Tamoxifen(20 mg/d) C: Tamoxifen(20 mg/d)	3 months	(a) The total effective rates (e) Side effects after treatment
Li, 2009	E1: 54 E2: 54 C: 54	20–60 years, Average Age: 37.5 years	–	E1: Ru-Pi-Xiao(15 tablets/d) + Tamoxifen(10 mg/d) E2: Ru-Pi-Xiao(15 tablets/d) C: Tamoxifen(10 mg/d)	1 menstrual cycle	(a) The total effective rates (e) Side effects after treatment
Zheng, 2011	E1: 123 E2: 124 C: 120	25–62 years, Average Age: 39.4 years	Average course: 11 month	E1: Ru-Pi-Xiao(18 tablets/d) + Tamoxifen(20 mg/d) E2: Ru-Pi-Xiao(18 tablets/d) C: Tamoxifen(20 mg/d)	3 menstrual cycle	(a) The total effective rates
Yang, 2006	E:198 C:58	E: 20–30 years (*n* = 45), 31–40 years (*n* = 69), 41–50 years (*n* = 75), 51–60 years (*n* = 9); C: 20–30 years (*n* = 13), 35–40 years (*n* = 25), 40–50 years (*n* = 17), 51–60 years (*n* = 3)	–	E: Ru-Pi-Xiao(24 g/d) + Tamoxifen(20 mg/d) C: Tamoxifen(20 mg/d)	3 menstrual cycle	(a) The total effective rates
Wang et al., 2004	E1: 86 E2: 48 C: 82	E1: 31.46 ± 8.32 years E2: 32.24 ± 8.56 years C: 32.68 ± 7.84 years	E1: 26 months E2: 16 months C: 22 months	E1: Ru-Pi-Xiao(18 tablets/d) + Tamoxifen(20 mg/d) E2: Ru-Pi-Xiao(18 tablets/d) C: Tamoxifen(20 mg/d)	3 menstrual cycle	(a) The total effective rates (e) Side effects after treatment
Xia, 2016	E:127 C:127	E: 41.5 ± 4.4 years C: 39.2 ± 4.2 years	E: 15.6 ± 0.7 months C: 17.1 ± 0.8 months	E: Ru-Pi-Xiao(6 g/d) + Tamoxifen(20 mg/d) C: Tamoxifen(20 mg/d)	3 months	(a) The total effective rates (e) Side effects after treatment

### Methodological quality

**Figure 2:**
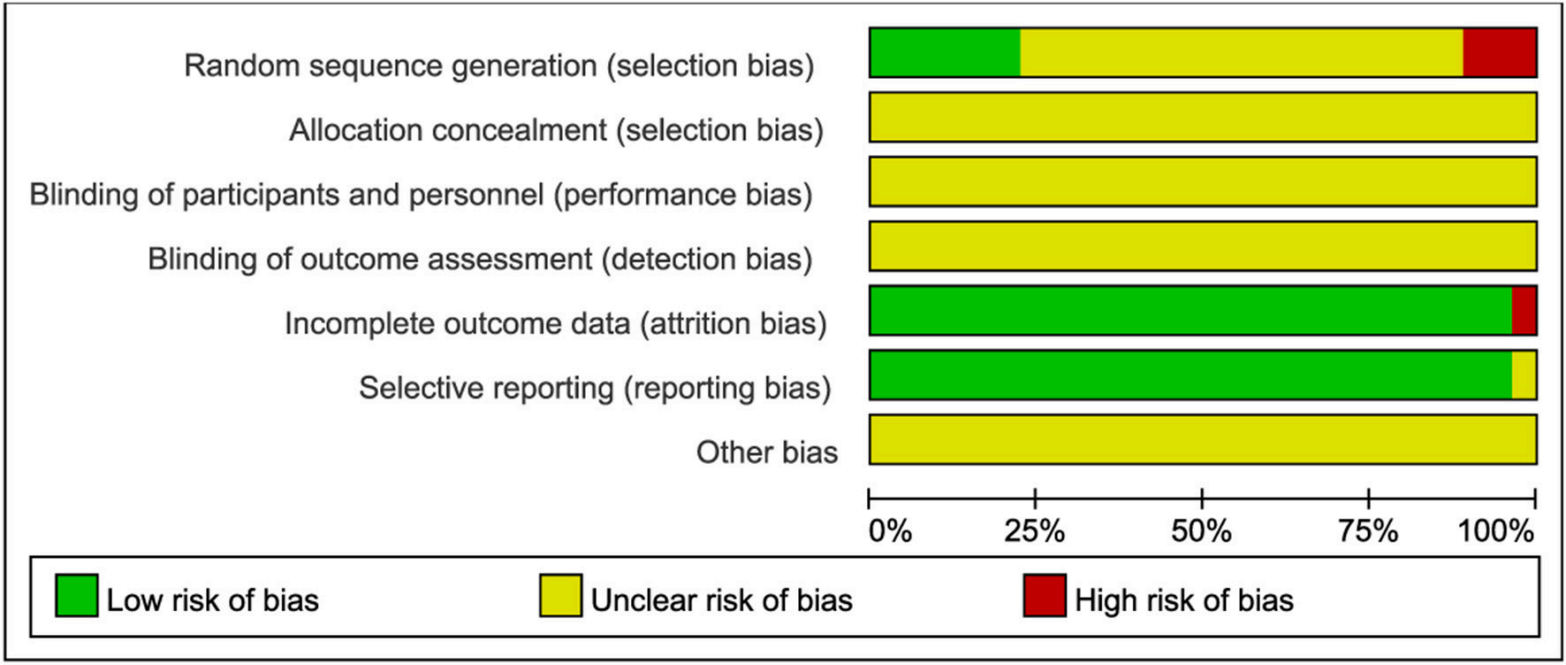
Risk of bias graph.

Among these 27 included articles, 18 studies (66.7%) mentioned the allocation sequence generation without showing the specific random method (Xia and Deng, 2001; Yang, 2006; Liu et al., 2008, 2013; Li, 2009; Zheng, 2011; Zhang et al., 2012; Wang, 2014, 2015; Xiao et al., 2014; Wang et al., 2015; Yin et al., 2015; Yue, 2015; Zhao, 2015; Kong and Huang, 2016; Ren, 2016; Xia, 2016; Zhu, 2016). In contrast, three articles used the random number table method (Liu et al., 2013; Wang et al., 2014; Liu, 2015), one article used the simple random sampling method (Ma and Xu, 2007), one article used the stratified sampling method (Cao, 2016), and one article used the method of randomized block (Yang, 2006), indicating that six articles (22.2%) ran a low risk of bias in random sequence generation. However, three articles (11.1%) generated the allocation sequence based on hospital or clinic record number, which should be judged as high risk (Wang, 2015; Huang, 2016; Xia, 2016). All studies provided completed outcome data, except one article (3.7%) missed the outcome data of progesterone level after drug treatment (Wang et al., 2015). The most common flaws were that all articles did not report the allocation concealment and blinding method. None of these studies clearly illustrated other bias. The risk of bias graph were shown in Figure 2.

### Meta-analysis

#### Total effective rates of “Ru-Pi-Xiao plus tamoxifen” vs. “tamoxifen” for MGH

**Figure 3:**
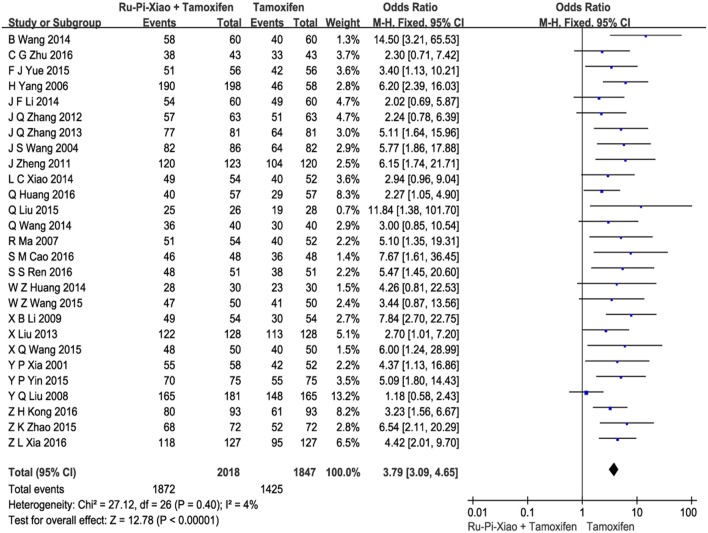
Forest plot of the total effective rates of “Ru-Pi-Xiao Plus Tamoxifen” vs. “Tamoxifen” for MGH. MGH, mammary gland hyperplasia. Study item displayed as first author with the publication year. I-squared and P are the criterion of the heterogeneity test, ♦ pooled mean difference, -■- mean difference, and 95% confidence interval. The total effective rates of specified drugs were defined as the incidence of events in which the patients experienced less pain in breast and had a decrease in size of breast lump by more than 1/3 after drug therapy.

Base on the “Guidelines for the Clinical Research on New TCM Drugs (1997)” published by the Ministry of Health P.R. China, patients who experienced less pain in breast and had a decrease in size of breast lump by more than 1/3 after drug therapy were considered to “be effective.” All the included trials compared clinical total effectiveness of “Ru-Pi-Xiao Plus Tamoxifen” vs. “Tamoxifen” for MGH (*n* = 3,865). Since the test for heterogeneity was statistically insignificant (*P* = 0.40, *I*^2^ = 4%), fixed effect model has been used in the meta-analysis. The total effective rate of “Ru-Pi-Xiao Plus Tamoxifen” was 92.76% (1,872 devided by 2018), whereas the total effective rate of “Tamoxifen” was 77.15% (1,425 devided by 1,847), indicating that “Ru-Pi-Xiao Plus Tamoxifen” might achieve a better pharmocological effect on MGH than “Tamoxifen.” The odds ratio for the improvement of MGH for “Ru-Pi-Xiao Plus Tamoxifen” treated vs. “Tamoxifen” treated was 3.79 (95% CI: 3.09–4.65; *P* < 0.00001), which achieved statistical significance (as shown in Figure 3).

### Total effective rates of “Ru-Pi-Xiao” vs. “tamoxifen” for MGH

**Figure 4:**
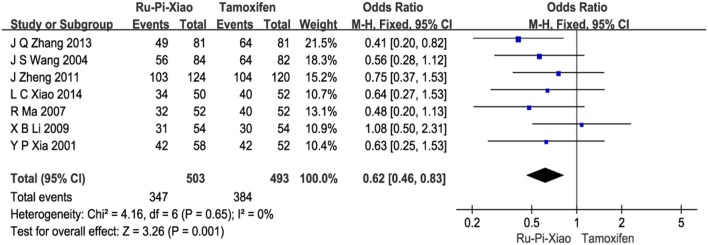
Forest plot of the total effective rates of “Ru-Pi-Xiao” vs. “Tamoxifen” for MGH. MGH, mammary gland hyperplasia. Study item displayed as first author with the publication year. I-squared and P are the criterion of the heterogeneity test, ♦ pooled mean difference, -■- mean difference, and 95% confidence interval. The total effective rates of specified drugs were defined as the incidence of events in which the patients experienced less pain in breast and had a decrease in size of breast lump by more than 1/3 after drug therapy.

Seven articles (*n* = 996) compared clinical total effectiveness of “Ru-Pi-Xiao” vs. “Tamoxifen” for MGH (Xia and Deng, 2001; Wang et al., 2004; Ma and Xu, 2007; Li, 2009; Zheng, 2011; Zhang et al., 2013; Xiao et al., 2014). Since the test for heterogeneity was statistically insignificant (*P* = 0.65, *I*^2^ = 0%), fixed effect model has been used in the meta-analysis. The total effective rate of “Ru-Pi-Xiao” was 68.99% (347 devided by 503), while the total effective rate of “Tamoxifen” was 77.89% (384 devided by 493), suggesting that “Ru-Pi-Xiao” might be slightly less effective than “Tamoxifen” in monotherapy. The odds ratio for the improvement of MGH for “Ru-Pi-Xiao” treated vs. “Tamoxifen” treated was 0.62 (95% CI: 0.46–0.83; *P* = 0.001), which achieved statistical significance (as shown in Figure 4).

### Level of plasma estradiol (E2) after treatment with “Ru-Pi-Xiao plus tamoxifen” vs. “tamoxifen”

**Figure 5:**
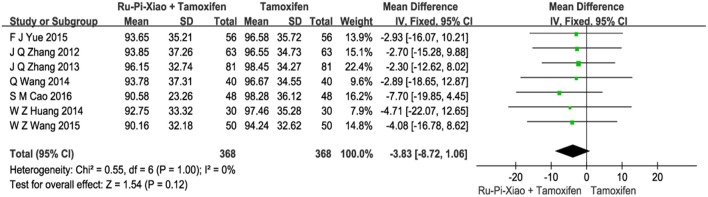
Forest plot of the level of “E2” after treatment with “Ru-Pi-Xiao Plus Tamoxifen” vs. “Tamoxifen” for MGH. E2, plasma estradiol; MGH, mammary gland hyperplasia. Study item displayed as first author with the publication year. I-squared and P are the criterion of the heterogeneity test, ♦ pooled mean difference, -■- mean difference, and 95% confidence interval.

Seven articles (*n* = 736) compared the level of plasma estradiol of “Ru-Pi-Xiao Plus Tamoxifen” vs. “Tamoxifen” after treatment (Zhang et al., 2012, 2013; Huang and Yi, 2014; Wang et al., 2014, 2015; Yue, 2015; Cao, 2016). Since the test for heterogeneity was insignificant statistically (*P* = 1.00, *I*^2^ = 0%), fixed effect model has been used in the meta-analysis. The level of **E2** treated with “Ru-Pi-Xiao Plus Tamoxifen” seemed to be lower than that of “Tamoxifen.” However, the mean differences for the level of **E2** for “Ru-Pi-Xiao Plus Tamoxifen” treated vs. “Tamoxifen” treated was −3.83 (95% CI: −8.72 to 1.06; *P* = 0.12), which was statistically insignificant (as shown in Figure 5).

### Level of progesterone (P) after treatment with “Ru-Pi-Xiao plus tamoxifen” vs. “tamoxifen”

**Figure 6:**
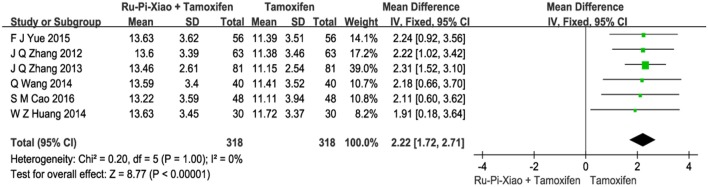
Forest plot of the level of “P” after treatment with “Ru-Pi-Xiao Plus Tamoxifen” vs. “Tamoxifen” for MGH. P, progesterone; MGH, mammary gland hyperplasia. Study item displayed as first author with the publication year. I-squared and P are the criterion of the heterogeneity test, ♦ pooled mean difference, -■- mean difference, and 95% confidence interval.

Six articles (*n* = 636) compared the level of progesterone of “Ru-Pi-Xiao Plus Tamoxifen” vs. “Tamoxifen” after treatment (Zhang et al., 2012, 2013; Huang and Yi, 2014; Wang et al., 2014; Yue, 2015; Cao, 2016). Since the test for heterogeneity was insignificant statistically (*P* = 1.00, *I*^2^ = 0%), fixed effect model has been used in the meta-analysis. The mean differences for the level of **P** for “Ru-Pi-Xiao Plus Tamoxifen” treated vs. “Tamoxifen” treated was 2.22 (95% CI: 1.72–2.71; *P* < 0.00001), which achieved statistical significance. Results have shown that the level of **P** treated with “Ru-Pi-Xiao Plus Tamoxifen” is statistically higher than that of “Tamoxifen” (as shown in Figure 6).

### Level of lutrophin (LH) after treatment with “Ru-Pi-Xiao plus tamoxifen” vs. “tamoxifen”

**Figure 7:**
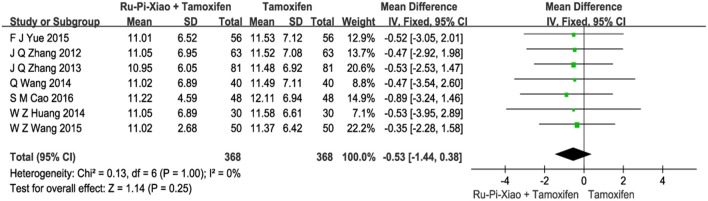
Forest plot of the level of “LH” after treatment with “Ru-Pi-Xiao Plus Tamoxifen” vs. “Tamoxifen” for MGH. LH, lutrophin; MGH, mammary gland hyperplasia. Study item displayed as first author with the publication year. I-squared and P are the criterion of the heterogeneity test, ♦ pooled mean difference, -■- mean difference, and 95% confidence interval.

Seven articles (*n* = 736) compared the level of progesterone of “Ru-Pi-Xiao Plus Tamoxifen” vs. “Tamoxifen” after treatment (Zhang et al., 2012, 2013; Huang and Yi, 2014; Wang et al., 2014, 2015; Yue, 2015; Cao, 2016). Since the test for heterogeneity was insignificant statistically (*P* = 1.00, *I*^2^ = 0%), fixed effect model has been used in the meta-analysis. The level of **LH** treated with “Ru-Pi-Xiao Plus Tamoxifen” seemed to be lower than that of “Tamoxifen.” However, the mean differences for the level of **LH** for “Ru-Pi-Xiao Plus Tamoxifen” treated vs. “Tamoxifen” treated was −0.53 (95% CI: −1.44 to 0.38; *P* = 0.25), which was statistically insignificant (as shown in Figure 7).

### Diameter of breast lumps after treatment with “Ru-Pi-Xiao plus tamoxifen” vs. “tamoxifen”

**Figure 8:**
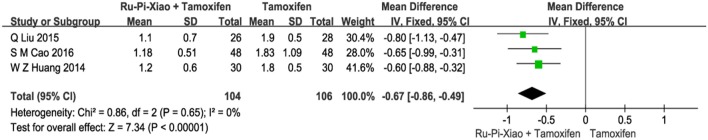
Forest plot of the diameter of breast lumps after treatment with “Ru-Pi-Xiao Plus Tamoxifen” vs. “Tamoxifen” for MGH. MGH, mammary gland hyperplasia. Study item displayed as first author with the publication year. I-squared and P are the criterion of the heterogeneity test, ♦ pooled mean difference, -■- mean difference, and 95% confidence interval.

Three articles (*n* = 210) compared the size of MGH patients' breast lumps of “Ru-Pi-Xiao Plus Tamoxifen” vs. “Tamoxifen” after treatment (Huang and Yi, 2014; Liu, 2015; Cao, 2016). The breast lump sizes of the participants were measured by the mammography X-ray examination. Since the test for heterogeneity was insignificant statistically (*P* = 0.65, *I*^2^ = 0%), fixed effect model has been used in the meta-analysis. The mean differences for the diameter of breast lumps for “Ru-Pi-Xiao Plus Tamoxifen” treated vs. “Tamoxifen” treated was −0.67 (95% CI: −0.86 to −0.49; *P* < 0.00001), which achieved statistical significance. Results have indicated that the diameter of breast lumps after treatment with “Ru-Pi-Xiao Plus Tamoxifen” is statistically smaller than that of “Tamoxifen” (as shown in Figure 8).

### Side effects after treatment with “Ru-Pi-Xiao plus tamoxifen” vs. “tamoxifen”

**Figure 9:**
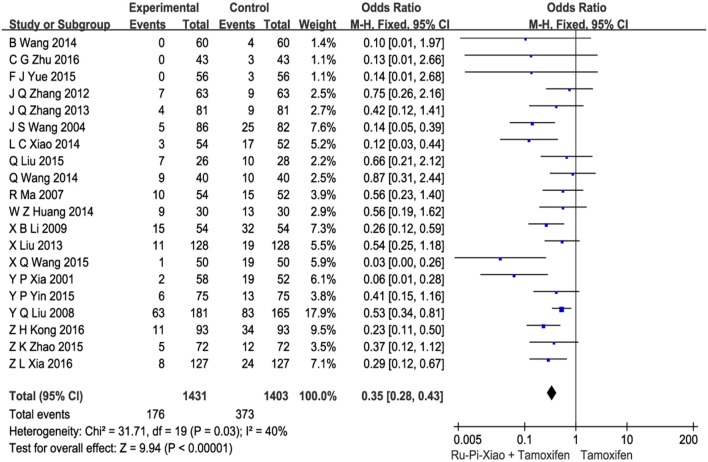
Forest plot of the overall incidence of drug adverse reactions after treatment with “Ru-Pi-Xiao Plus Tamoxifen” vs. “Tamoxifen” for MGH. MGH, mammary gland hyperplasia. Study item displayed as first author with the publication year. I-squared and P are the criterion of the heterogeneity test, ♦ pooled mean difference, -■- mean difference, and 95% confidence interval.

**Figure 10:**
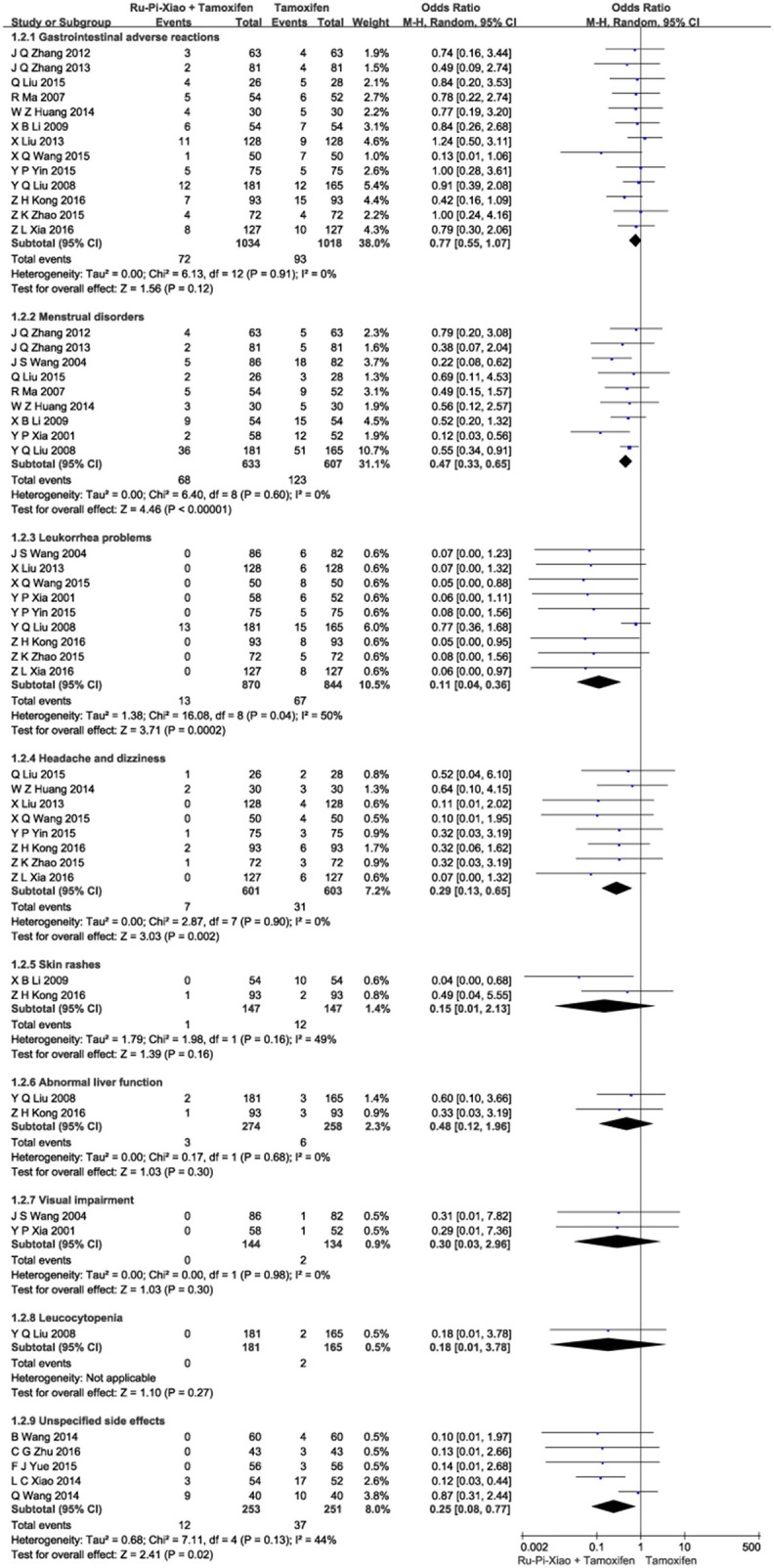
Forest plot of the rates of different types of side effects after treatment with “Ru-Pi-Xiao Plus Tamoxifen” vs. “Tamoxifen” for MGH. MGH, mammary gland hyperplasia. Study item displayed as first author with the publication year. Subgroups were divided by the characteristics of different drug adverse reactions, including: (1) gastrointestinal adverse reactions; (2) menstrual disorders; (3) leukorrhea problems; (4) headache and dizziness; (5) skin rashes; (6) abnormal liver function; (7) visual impairment; (8) leucocytopenia; (9) unspecified side effects. I-squared and P are the criterion of the heterogeneity test, ♦ pooled mean difference, -■- mean difference, and 95% confidence interval.

This part of meta-analysis included at least eight kinds of side effects such as gastrointestinal adverse reactions, menstrual disorders, leukorrhea problems, headache and dizziness, skin rashes, abnormal liver function, visual impairment, and leucocytopenia. Twenty articles (*n* = 2,834) compared the side effects of “Ru-Pi-Xiao Plus Tamoxifen” vs. “Tamoxifen” for MGH (Xia and Deng, 2001; Wang et al., 2004, 2014; Ma and Xu, 2007; Liu et al., 2008, 2013; Li, 2009; Zhang et al., 2012, 2013; Huang and Yi, 2014; Wang, 2014, 2015; Xiao et al., 2014; Liu, 2015; Yin et al., 2015; Yue, 2015; Zhao, 2015; Kong and Huang, 2016; Xia, 2016; Zhu, 2016). Since the test for heterogeneity was insignificant statistically (*I*^2^ = 40%), fixed effect model has been used in the meta-analysis. The overall incidence of drug adverse reactions in “Ru-Pi-Xiao Plus Tamoxifen” group was 12.30% (176 devided by 1,431), whereas the overall incidence of drug adverse reactions in “Tamoxifen” group was 26.58% (373 devided by 1,403), indicating that the combination of Ru-Pi-Xiao and tamoxifen might caused fewer side effects than tamoxifen alone in the treatment of MGH. The odds ratio for side effects of drug therapy for “Ru-Pi-Xiao Plus Tamoxifen” treated vs. “Tamoxifen” treated was 0.35 (95% CI: 0.28–0.43; *P* < 0.00001), which achieved statistical significance (as shown in Figure 9). The subgroup analysis of the rates of different types of side effects after treatment with “Ru-Pi-Xiao Plus Tamoxifen” vs. “Tamoxifen” for MGH was also displayed in Figure 10, showing more details about drug safety with the application of Ru-Pi-Xiao and tamoxifen. Results have indicated that the incidences of specified drug adverse reactions such as menstrual disorders, headache and dizziness, and leukorrhea problems treated by “Ru-Pi-Xiao Plus Tamoxifen” are statistically lower than that of “Tamoxifen.”

## Discussion

### Summary of findings

Our meta-analysis of the current literature showed that one of the most commonly used Chinese Medicine for treating MGH named “Ru-Pi-Xiao” can significantly improve its symptoms and relative hormonal parameters in MGH patients. The number of trials in the extant systematic reviews conducting on MGH were insufficient, and these papers only focused on the total effective rates for treating MGH, without discussing and evaluating the variation in objective measures. In this review, we gathered information on 4,368 subjects from 27 articles and combined all of the evidences from these studies to investigate not only total effective rates of “Ru-Pi-Xiao Plus Tamoxifen,” “Ru-Pi-Xiao,” and “Tamoxifen” treated, but also variation in the level of plasma estradiol, progesterone, lutrophin, and the size of breast lump. It should be noted that 12-week treatment cycle was the most common intervention.

To evaluate total effective rates in experimental and control groups after drug treatment, those participants who experienced less pain in breast and had a decrease in size of breast lump by more than 1/3 were considered to “be effective.” Although the response rate of MGH patients treated by “Ru-Pi-Xiao” alone was lower than that of “Tamoxifen,” the combination of Ru-Pi-Xiao and tamoxifen exhibited better therapeutic effects on the total effective rates than “tamoxifen” treated alone, indicating that the combined use of Ru-Pi-Xiao and tamoxifen might be a better choice for clinical treatment of MGH.

Six to Seven included studies have reported that both “Ru-Pi-Xiao Plus Tamoxifen” and “Tamoxifen” treatment could decrease the hormonal level of plasma estradiol and lutrophin, and increase progesterone concentration in MGH patients. However, our meta-analysis revealed that after treatment, the difference in the plasma level of estradiol and lutrophin was not statistically significant between the experimental group (“Ru-Pi-Xiao Plus Tamoxifen”) and the control group (“Tamoxifen” alone). In contrast, the level of progesterone after “Ru-Pi-Xiao Plus Tamoxifen” treatment was obviously higher than that in the control group, which was statistically significant. These results might provide an information regarding the pharmacodynamic mechanism that “Ru-Pi-Xiao Plus Tamoxifen” treatment might return progesterone concentration back toward normal more rapidly than that of “Tamoxifen” alone, so as to achieve a more robust biological response to relieve pathogenetic condition in MGH patients.

Our meta-analysis also found that the incidence of adverse drug reaction in the experimental group (“Ru-Pi-Xiao Plus Tamoxifen”) is significantly lower than that of the control group (“Tamoxifen” alone). As shown in Figures 9, 10, a total of 373 participants (26.6%) in the control group (*n* = 1,403) suffered from side effects, including gastrointestinal adverse reactions (93 patients, 6.63%), menstrual disorders (123 patients, 8.77%), leukorrhea problems (67 patients, 4.78%), headache and dizziness (31 patients, 2.21%), skin rashes (12 patients, 0.86%), abnormal liver function (6 patients, 0.43%), visual impairment (2 patients, 0.14%), leucocytopenia (2 patients, 0.14%), and cases for unspecified reasons (37 patients). Meanwhile, a total of 176 subjects (12.3%) in the experimental group (*n* = 1,431) suffered from side effects, including gastrointestinal adverse reactions (72 patients, 5.03%), menstrual disorders (68 patients, 4.75%), leukorrhea problems (13 patients, 0.91%), headache and dizziness (7 patients, 0.49%), skin rashes (1 patients, 0.07%), abnormal liver function (3 patients, 0.21%), and cases for unspecified reasons (12 patients). These data have indicated that gastrointestinal adverse reactions, menstrual disorders, leukorrhea problems, headache and dizziness might be the principal manifestations of side effects. The combination of Ru-Pi-Xiao and tamoxifen could decrease the incidence of these reactions, especially the risk of menstrual disorders and leukorrhea problems, which might be caused by sex hormone abnormality.

Although studies have demonstrated that the combination of Ru-Pi-Xiao and tamoxifen could represent much better therapeutic effects and drug safety on MGH patients than tamoxifen mainly through modulating the expression level of progesterone, these results remain inconclusive and require further investigation.

### Strengths and limitations

The included articles were searched from a wide range of electronic databases (e.g., PubMed, EMBASE, Wiley Online Library, and Springer link). Considering that Ru-Pi-Xiao is a kind of Chinese medicine, we found relevant information from the largest Chinese information databases (e.g., China Knowledge Resource Integrated database, SinoMed database, Wanfang database, and VIP database). This meta-analysis was the first meta-analysis to evaluate the effects of Ru-Pi-Xiao and tamoxifen on the improvement of plasma estradiol, progesterone, lutrophin, and the size of breast lump. Furthermore, 20 articles of the included studies (74.1%) were published over the last five years (from 2012 to 2016). To reduce bias and transcription errors, six authors independently performed study selection, data extraction, and quality assessment processes.

However, our meta-analysis had several limitations. First, the selected articles were all published in China, and related studies in other countries remained unclear. It should be of great help to support the international use of this drug combination, if RCTs in other countries' patient populations can also be performed. Second, although all included articles claimed to be RCTs, 3 articles (11.1%) ran a high risk of bias in random sequence generation, and 1 article (3.7%) ran a high risk of bias in incomplete outcome data. Eighteen studies (66.7%) only mentioned allocation sequence generation without showing the specific random method, and all articles did not report the allocation concealment and blinding method. The low quality of the articles included in this review might lead to some overestimation of the overall efficacy of Ru-Pi-Xiao and tamoxifen combination in comparison to tamoxifen. Therefore, RCTs with high quality are still required to clarify this issue. Third, whether the MGH patients were histologically verified may have a closed relationship with the emergence of breast cancer. Unfortunately, the histological subtypes of MGH patients in the included RCTs were not mentioned at all. Thus we could not sort the participants by histology. Fourth, we did not search for any unpublished trials. Fifth, the results of meta-analysis may be affected by the dosage of Ru-Pi-Xiao and tamoxifen. Eligible studies, however, employed different dosing parameters, especially the variation in the dosage of Ru-Pi-Xiao. Seventeen included articles (63.0%) used “tablet,” “pill,” or “bag” as the unit mass of Ru-Pi-Xiao, the exact doses of which were undescribed (Xia and Deng, 2001; Wang et al., 2004, 2014, 2015; Ma and Xu, 2007; Liu et al., 2008; Li, 2009; Zheng, 2011; Zhang et al., 2012; Wang, 2014; Liu, 2015; Yue, 2015; Cao, 2016; Huang, 2016; Kong and Huang, 2016; Ren, 2016; Zhu, 2016). In 10 other reports claiming the exact doses of drugs, half of them set the dose value of Ru-Pi-Xiao to 6 g/d in experimental group (Liu et al., 2013; Wang, 2015; Yin et al., 2015; Zhao, 2015; Xia, 2016), suggesting that patients administered with 6 g/d of Ru-Pi-Xiao plus 20 mg/d of tamoxifen might be recommendable. Sixth, the age of participants (from 18 to 60 years) and the duration of MGH (from 7 days to 12 years) were varied over a wide range in these RCTS, and the therapeutic effects on MGH patients with different age and duration time need further evaluation. Seventh, RCTs showing decreasing the size of breast lump included in our meta-analysis had small sample sizes. Thus, future meta-analyses including more large-scale RCTs are required to further prove this effect. Eighth, 7 included articles (25.9%) made no mention of drug adverse reactions (Yang, 2006; Zheng, 2011; Li, 2014; Wang et al., 2015; Cao, 2016; Huang, 2016; Ren, 2016), and 5 included articles (18.5%) did not describe the types and characteristics of drug adverse reactions (Wang, 2014; Wang et al., 2014; Xiao et al., 2014; Yue, 2015; Zhu, 2016). Due to insufficient descriptions, data for adverse reactions just included gastrointestinal adverse reactions, menstrual disorders, leukorrhea problems, headache and dizziness, skin rashes, abnormal liver function, visual impairment, and leucocytopenia. A more rational approach for the evaluation of adverse drug reactions from the treatments of Ru-Pi-Xiao and tamoxifen will be needed in the future.

## Conclusion

Since MGH might increase the risk of breast cancer, its prevention and treatment is believed to be an effective means for breast cancer prevention. This meta-analysis was probably the first systematic review to determine the effects of Chinese medicine for treating MGH by investigating not only total effective rates, but also the variation in relative hormonal parameters and pathological characteristics. Although this review exists certain limitations, it might have proved that the combination of Ru-Pi-Xiao and tamoxifen can exhibit better therapeutic effects against MGH while ameliorating side effects. Based on the results of this study, we proposed the hypothesis regarding the pharmacodynamic mechanism that “Ru-Pi-Xiao Plus Tamoxifen” drug therapy might return progesterone concentration back toward normal more rapidly than that of “Tamoxifen” alone, so as to relieve the pathogenetic condition of MGH patients to a greater extent. More additional large-scale, well-designed trials are urgently required to confirm these results.

## Author contributions

H-TL, H-HL, and Y-XY: Did the literature database search, data collection, and data extraction; PZ, R-LW, and J-YL: Performed data analysis; H-HL, YY, S-NL, and YZ: Contributed to the rationalization of the results; H-TL, L-QQ, X-LZ, and TW: Wrote the manuscript; S-ZW, KL, and P-YL: Gave advice on preparing the writing; The topic was conceptualized by L-QQ, H-HL, YY, S-NL, and YZ.

### Conflict of interest statement

The authors declare that the research was conducted in the absence of any commercial or financial relationships that could be construed as a potential conflict of interest.
